# Structural validation of novel bileaflet mechanical heart valve hinge mechanism

**DOI:** 10.1177/09544119251403379

**Published:** 2025-12-23

**Authors:** Dylan Goode, Usama Ishtiaq, Dahlia Mohammadi, Hadi Mohammadi

**Affiliations:** 1 The Heart Valve Performance Laboratory, School of Engineering, Faculty of Applied Science, University of British Columbia, Kelowna, Canada; 2 Department of Mechanical, Mechatronics and Manufacturing Engineering, University of Engineering and Technology Lahore, Punjab, Pakistan

**Keywords:** heart valve, prosthetic heart valves, mechanical heart valves, cardiovascular engineering, medical devices, finite element analysis

## Abstract

The structural performance of hinges in mechanical heart valves (MHVs) is essential for durability and reliability. This study evaluates the iValve, a novel bileaflet MHV, using advanced finite element method (FEM) simulations to assess its hinge design under physiological and supra-physiological conditions. The hinge design aims to minimize stress concentrations, reduce wear, and enhance durability compared to conventional valves. A detailed 3D FEM model, incorporating precise hinge geometry, was developed to analyze stress distribution, deformation, and potential failure zones. While our study uses a quasi-static finite element approach, and thus does not capture full dynamic or fluid-structure interactions, it evaluates peak physiological loading conditions representative of the cardiac cycle. The results show a lower and more uniform stress distribution in the iValve compared to conventional bileaflet MHVs, suggesting reduced stress concentrations and potentially improved fatigue life. The model was validated against experimental data from in vitro flow simulators, ensuring accurate representation of the hemodynamic forces during the cardiac cycle. Results show that the iValve’s hinge design achieves superior stress distribution with significantly lower peak von Mises stresses than traditional designs. Optimized materials and geometric features reduce the risk of fatigue and wear, while high-cycle fatigue simulations confirmed minimal deformation, demonstrating suitability for extended use. This study highlights the role of FEM in advancing MHV design by balancing mechanical performance with physiological compatibility. The iValve addresses hinge failure and thrombus risks, offering a durable, anticoagulation-free solution.

## Introduction

The development of mechanical heart valves began on September 11th, 1952, when Dr. Charles Hufnagel implanted the first ball and cage valve into a patient.^1^ Significant design advancements in the 1950s to ‘70s led to the current bileaflet mechanical heart valve (BMHV) design. BMHVs are the only mechanical valves approved by the FDA, Health Canada, and CE for implantation, with no major design changes since the introduction of the St. Jude Medical (SJM) Regent BMHV in 1976 (now owned by Abbott Laboratories).^2^ Although new three-leaflet designs are in clinical trials,^3^ the bileaflet design has remained largely unchanged, with only minor modifications.

Conventional BMHVs have two leaflets that create three orifices, failing to mimic the native valve’s behavior. They require lifelong anticoagulation therapy to prevent thrombosis due to non-physiological shear stress in the hinge area and spikes in backflow velocity.^4,5^ This anticoagulation therapy increases the risk of severe bleeding and blood clots if levels are not properly maintained.^6^ The hinge mechanism is crucial to BMHV performance, with its geometry, influenced by the leaflet design, significantly impacting hemodynamics.

The Medtronic Open Pivot BMHV uses spherical convex pivot guides on the inner radius of the orifice, eliminating recesses and allowing the leaflets to rotate on protruding spheres.^7^ This design exposes the hinge area fully, enhancing the washing effect and enabling the leaflets to open to 85° with rapid closure, reducing regurgitation and noise.^7^

The Corcym Carbomedics BMHV features a butterfly recess with pronounced nodal side contact of the leaflet appendage to the housing socket. The leaflets form a 25° angle when closed and open to 78°.^8^

The On-X BMHV includes a butterfly recess that allows the leaflets to open to 90° with a 50° total travel angle, facilitated by a closing ramp that shifts the leaflets 6° during closure initiation due to reversed blood flow drag forces.^9^

The SJM/Abbott Regent BMHV also uses a butterfly recess hinge near the central axis of the housing. Depending on valve size, the leaflets meet the housing at a 30°−35° angle when closed and open to 85°, resulting in a 50°−55° travel angle.^10,11^

**Figure 1.**
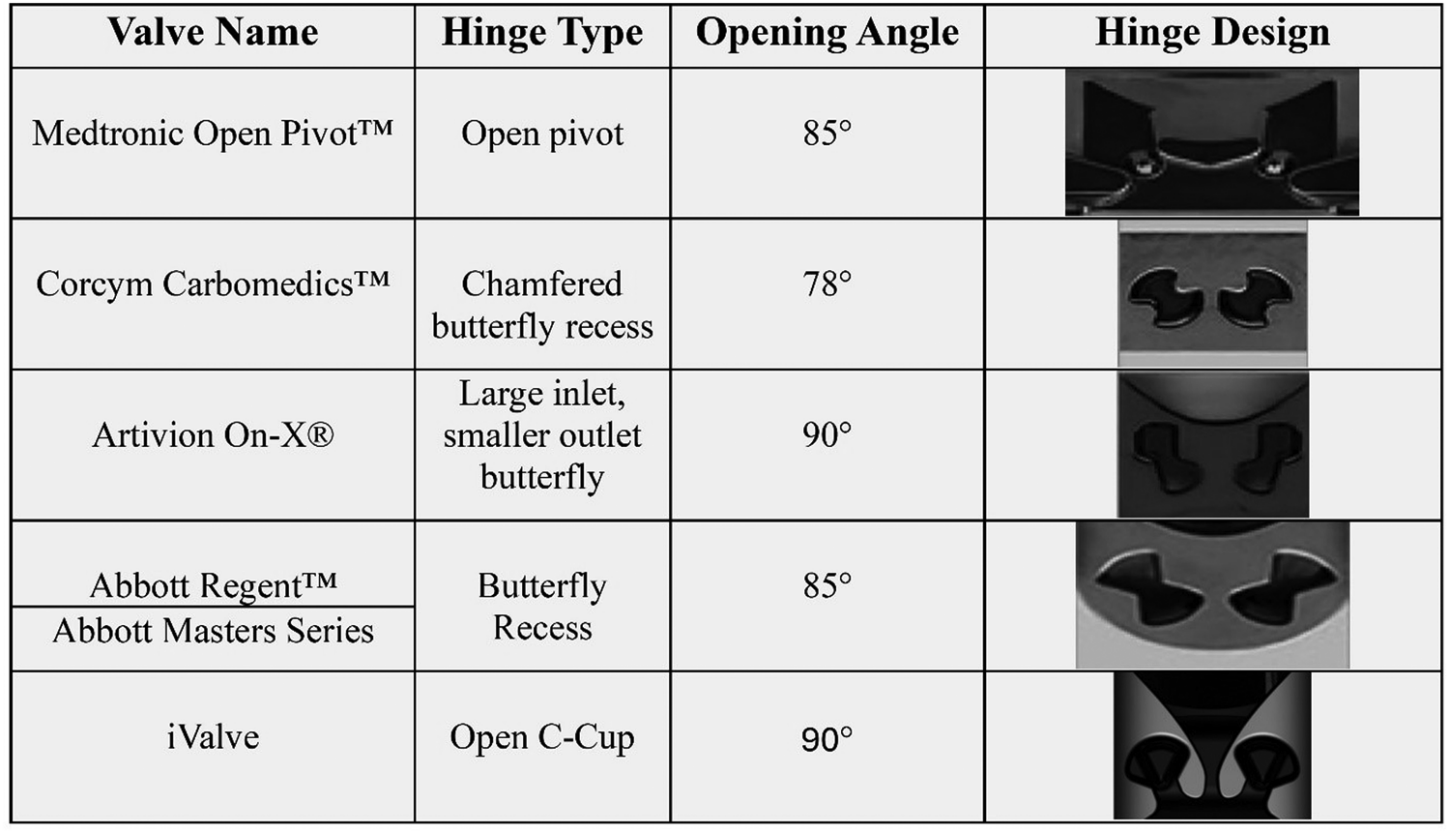
Conventional bileaflet mechanical heart valve (BMHV) hinge designs, covering commercial names, hinge mechanisms, and opening angles, with visuals highlighting their structure, function, and impact on performance. Adapted from Ref.^12^

All conventional BMHVs (Figure 1) emphasize hinge washing during the closed phase, except for the Medtronic Open Pivot BMHV, which allows washing during forward flow. This work introduces a novel BMHV hinge design aimed at achieving washing during forward flow, though it requires structural validation. The structural performance of these designs will be assessed through finite element analysis (FEA).

## Methods

The following sections describe the novel BMHV (iValve) design and the setup for the FEA of BMHV hinges.

### iValve design

The iValve changes the leaflet and hinge design compared to conventional BMHVs. While most BMHVs feature straight leaflets that create three orifices when fully open, the iValve’s rounded, eye-shaped leaflets allow for a single central orifice. The elliptic leaflets, inspired by research showing the SJM/Abbott Regent BMHV’s having improved hemodynamics when an ovality is applied,^12^ move similarly to an eyelid, hence the name iValve. The saddle-shaped housing of the iValve contrasts with the circular geometry of typical BMHVs, accommodating this unique leaflet movement.

**Figure 2.**
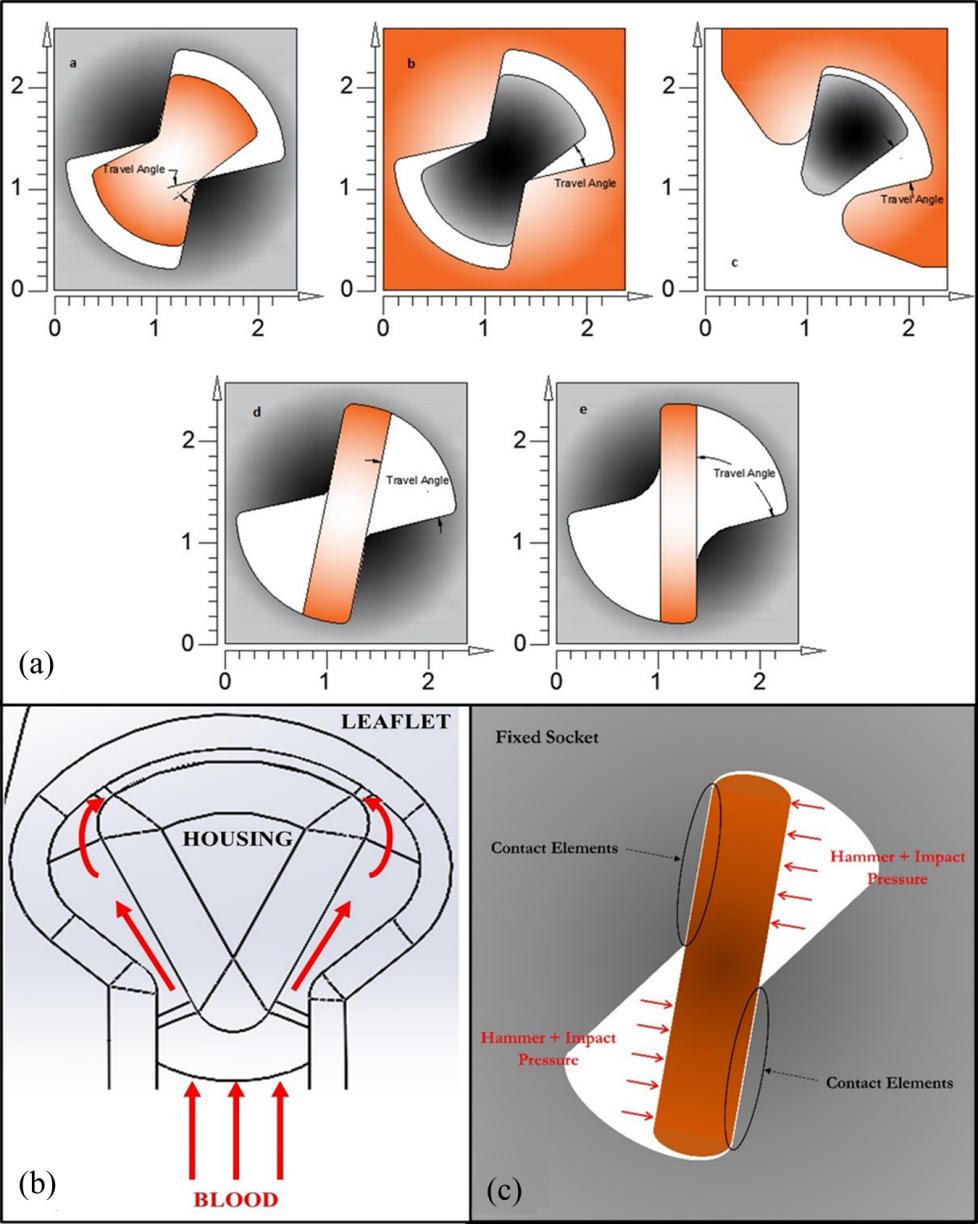
(a) Visualization of proposed hinge designs and standard BMHV hinge designs. (a), (b) and (c) are the designs proposed for the hinges of the iValve. (d) and (e) are the concepts used to design hinges in SJM/Abbott and On-X valves. The shade of gray indicates the housing, and the shade of orange indicates the leaflets. Opening angles for all models have been shown. (b) Design concept of iValve open hinge. The theorized washing effect of stagnant blood elements is displayed during the lower shear forward flow phase. (c) Example boundary conditions for two-dimensional finite element analysis.

The iValve’s hinge mechanism also differs from conventional BMHVs, which generally use a butterfly hinge socket. The iValve achieves a 90° opening angle, parallel to forward flow. The iValve’s open hinge design features a pie-shaped protrusion seated into a C-shaped open socket on the leaflet (Figure 2(b)). This open hinge concept aims to wash stagnant blood elements during the lower shear forward flow phase,^13^ unlike conventional BMHVs that wash their hinges during the high shear closed phase.^14^ The high shear rates in conventional valves, necessary to prevent thrombosis, are associated with hemolysis and coagulation initiation.^15^ The iValve seeks to eliminate the need for high-shear hinge washing by facilitating blood element removal during the lower-shear forward flow phase. By lower shear forward flow phase, we refer to the period of antegrade (forward) blood flow through the valve, whether in the aortic or mitral position, during which shear stresses are relatively low compared to peak flow conditions. This phase is defined by the direction and nature of flow (i.e. antegrade), rather than strictly by systole or diastole, as both aortic and mitral valves experience forward flow at different points in the cardiac cycle. Additional hinge designs for the iValve are explored in the structural hinge analysis.

### Two-dimensional structural hinge analysis setup

The primary novelty of the iValve lies in its hinge design, which reduces stress concentrations and improves durability. While the leaflet geometry has been refined for better hemodynamics, it serves to complement the hinge innovation rather than constitute a separate advancement. FEM and experimental results confirm that performance gains, lower stresses and enhanced fatigue resistance, stem mainly from the hinge.

The SJM/Abbott Regent BMHV and On-X BMHV (Figure 2(a)) were selected for comparison with the iValve in a structural hinge analysis. This study evaluates three hinge design concepts:

1. **Model 1:** A bowtie-shaped cavity socket on the housing with a bowtie-shaped leaflet appendage. This design reduces the hinge’s travel angle and impact force. However, washing is suboptimal because the cavity is hidden behind the leaflet.
2. **Model 2:** A bowtie-shaped cavity socket on the leaflets, with a bowtie-shaped appendage extending from the housing. This design improves hinge exposure to the bloodstream, enhancing washing efficiency.
3. **Model 3:** An improved version of Model 2, featuring a half-bowtie-shaped socket and appendage. The socket has a “C” shape (open loop) rather than an “O” shape (closed loop), allowing direct blood flow into the hinge cavity, which is expected to maximize washing during systole.

The hinges experience cyclic loading with each heartbeat, with the worst-case scenario occurring when the valve is fully closed during diastole. This phase involves two pressures: hammer and diastolic. Using a numerical approach based on previous studies,^16^ the hammer pressure is calculated as 27.75 mmHg (3.7 kPa). The heart rate and cardiac output were set at 72 BPM and 6 l/min, respectively. Diastolic pressure is assumed to be 80 mmHg (10.67 kPa) during the closing phase. The resulting maximum impact forces at the hinges range from 50 to 90 N. These forces were applied to a finite element model of the hinge areas, with boundary conditions illustrated in Figure 2(c). In the FEA, the socket was treated as fixed, and the appendage was secured to the socket using contact elements. The combined hammer and impact pressures were applied as a couple to the non-contact side of the appendage. A 2D plane strain FEA was performed.

### Three-dimensional structural hinge analysis setup

**Figure 3.**
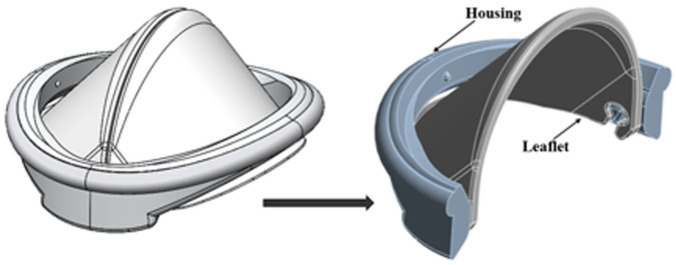
3D iValve geometry designed for finite element analysis to evaluate structural integrity, fluid dynamics, and mechanical performance under physiological conditions.

For the 3D structural analysis, Ansys Static Structural software was employed. To optimize computational efficiency, the housing of the iValve was bisected, allowing for the application of a symmetric boundary condition and reducing computational time. The geometric configuration used in the analysis is depicted in Figure 3, where 50% of the housing and a single leaflet of the iValve were modeled for the simulation.

Two materials were selected for analysis in this study: Pure low temperature isotropic pyrolytic carbon (LTI PyC) and silicon-alloyed LTI PyC. LTI PyC is a gold standard for BMHVs, with pure LTI PyC being utilized in the On-X BMHV, while the silicon-alloyed LTI PyC is seen in the SJM/Abbott BMHV. Both materials under evaluation are elastic models with differing mechanical properties. The detailed properties of both materials are presented in Table 1.

**Table 1.** Pure PyC and silicon-alloyed LTI PyC material properties.^15,16^

Material property	Pure LTI PyC	Si alloyed LTI PyC
Density (g/cm^3^)	1.93	2.12
Young’s modulus (GPa)	29.4	30.5
Poisson’s ratio	0.223	0.236
Bulk modulus (GPa)	17.69	19.2
Shear modulus (GPa)	12.02	12.34
Coefficient of thermal expansion (CTE; 10^6^ cm/cm°C)	6.5	6.1

**Figure 4.**
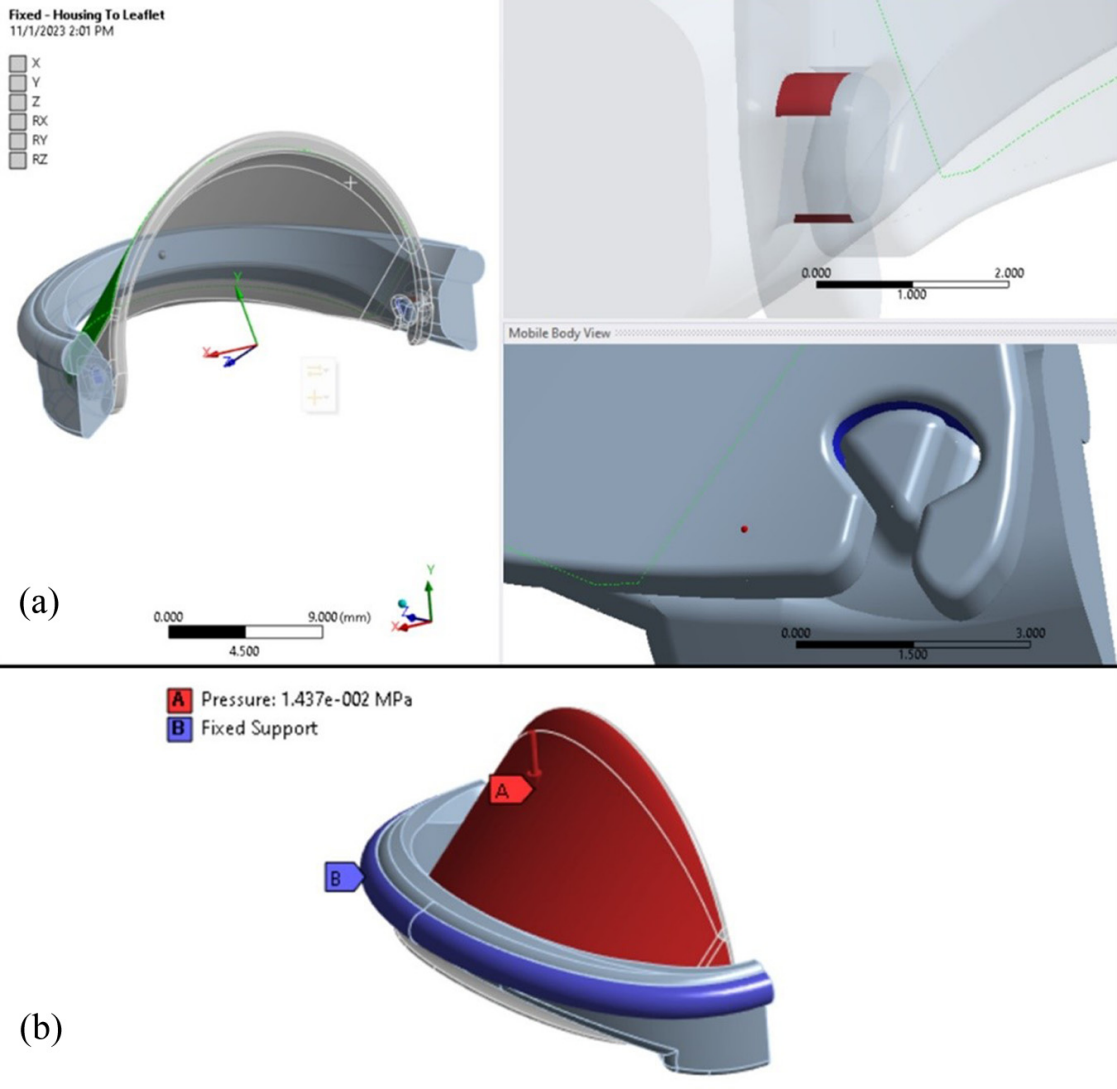
3D finite element analysis setup includes: (a) Visualization of the contact region in the iValve hinge area, highlighting interaction dynamics including a transparent view of the hinge mechanism to visualize the contact region between housing and leaflet (green surface denotes top surface of leaflet), and (b) Illustration of boundary conditions applied, detailing constraints and loads for accurate finite element analysis simulations.

Both bonded contact regions with shared topology and fixed joints were analyzed during the structural evaluation. After reviewing the results, the fixed joint configuration was chosen for further finite element analysis of the iValve. The fixed joint constrains all six degrees of freedom, making it easier to accurately analyze deformation and stress behavior, particularly with smaller mesh sizes. The use of a fixed joint between the housing and leaflet simulates a worst-case scenario, ensuring a conservative assessment of the valve’s performance. In a more realistic situation with leaflet motion and the same applied pressure, the resulting stresses would likely be less severe. Moreover, when utilizing bonded contact regions, stress tends to accumulate within the elements at smaller mesh sizes, eventually leading to the failure of the bonded contact. A visual representation of the contact region is shown in Figure 4(a).

In our quasi-static FE analysis, we applied a pressure load corresponding to peak diastolic pressure (approximately 80 mmHg), as this represents the primary sustained load on the closed leaflets during diastole. However, the most severe mechanical loading actually occurs at the moment of valve closure due to the water-hammer effect, an abrupt pressure spike caused by the rapid deceleration of blood flow. This transient impact load can significantly exceed steady diastolic pressure and is a critical contributor to stress concentrations and potential fatigue damage. To account for this, our model includes an additional impact (hammer) pressure component, estimated based on established hemodynamic models of bileaflet valve closure. The combined loading condition, diastolic pressure plus hammer pressure, was used to simulate the worst-case mechanical scenario for the leaflets and hinges. The summation of the hammer pressure, derived from a previous study,^16^ and the diastolic pressure equal to 14.37 kPa and is applied to the leaflet in the downward direction to simulate the physiological loading conditions. To restrict movement, a fixed support is applied at the suture ring, ensuring the housing remains stationary throughout the analysis. The ambient temperature for the simulation is set to 37°C, reflecting normal human body temperature. Additionally, a fixed joint is utilized between the housing and the leaflet to replicate a rigid connection, allowing for a conservative assessment of the valve’s structural response under these conditions. The applied load to the iValve geometry for the finite element analysis can be view in Figure 4(b).

### Mesh independence study

The analysis was conducted using COMSOL Multiphysics^®^. A mesh independence study determined the optimal mesh density for the contact zones, with linear first-order elements used in all models. About 10% of the elements were contact elements. To ensure the reliability of our results, we performed a systematic mesh convergence analysis using both 2D and 3D models. Specifically, the final 2D model employed 10,994 quadratic triangular elements, while the 3D model utilized 15,734 quadratic tetrahedral elements with refined seeding around contact regions to accurately capture stress gradients. The convergence study evaluated maximum von Mises stress in the high-stress zones as a function of element count and size. Mesh refinement was incrementally increased until the change in peak stress between successive refinements was less than 2%, confirming that the solution had effectively converged. The selected mesh densities represent the optimal balance between computational efficiency and accuracy for resolving contact stresses without unnecessary resource expenditure. In this study, the hinges of a BMHV and associated components were analyzed under physiologically relevant conditions to determine the optimal mesh density for capturing critical stress distributions and deformations while maintaining computational efficiency. The study began by defining a coarse initial mesh for the valve hinges, with subsequent refinements applied incrementally to achieve finer resolutions. The primary goal was to assess the convergence of key parameters such as von Mises stress, deformation, and contact pressure at the hinge interfaces. Physiological loading conditions were simulated, including representative flow forces and pressure gradients mimicking the cardiac cycle. By comparing the simulation outputs across mesh refinements, it was possible to evaluate how variations in element size affected the accuracy and stability of the results. Initially, the coarse mesh yielded noticeable variations in stress distribution, particularly at regions with high stress gradients, such as the hinge contact surfaces. These discrepancies suggested under-resolution of critical areas. As the mesh density increased, the differences in stress and displacement values between successive refinements diminished. Beyond a certain threshold mesh density, the results converged, indicating that the solution had become independent of the mesh size. This threshold marked the optimal balance between computational effort and result accuracy. The final results of the mesh independency study confirmed the robustness of the FE model. The identified optimal mesh density provided a reliable framework for analyzing the mechanical performance of the valve hinges, ensuring that predictions of stress, deformation, and contact behavior were both accurate and computationally efficient.

## Results and discussion

The following sections will detail the results from the 2D plane strain FEA, along with the 3D FEA.

### Two-dimensional structural hinge analysis results and discussion

**Figure 5.**
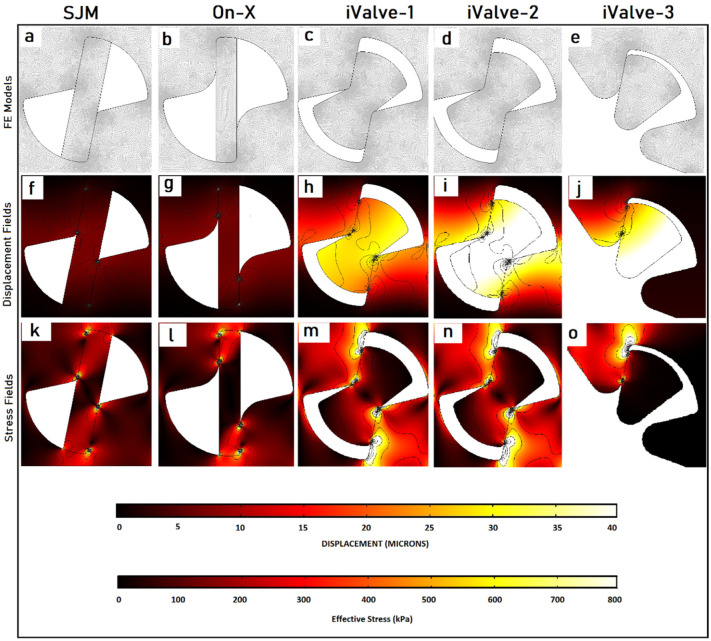
Finite element analysis (FEA) of hinges under impact loading. Panels (a–e) show the mesh models; (f–j) display the corresponding displacement fields (in micrometers, μm); and (k–o) illustrate the stress distributions (in kilopascals, kPa) around the hinges. Specifically, panels (f and k) present results for the SJM/Abbott valve, (g and l) for the On-X valve, and the remaining panels (h–j and m–o) correspond to the three proposed iValve designs introduced in this study.

The displacement and stress fields in the hinge areas for all models are displayed in Figure 5(f) to (o). The results indicate that in conventional models, stress is concentrated in a small area near the contact zone (Figure 5(k) and (l)), with the corresponding deformation shown in Figure 5(f) and 5(g). In contrast, the proposed designs distribute stress over larger areas (Figure 5(m)–(o)), significantly reducing maximum stress values. The corresponding displacement fields are shown in Figure 5(h) to (j), with the proposed designs exhibiting greater displacement due to the larger appendages. Lower stress levels in the proposed designs suggest a longer lifespan, even though direct fatigue analysis was not conducted.^17^ This analysis primarily aimed to identify stress concentrations and distribution in each hinge model. The improved stress distribution in the proposed models is promising for enhancing BMHV structural performance, validating the iValve’s open hinge concept, and supporting further investigation of its washing effects.

### Three-dimensional structural hinge analysis results and discussion

**Figure 6.**
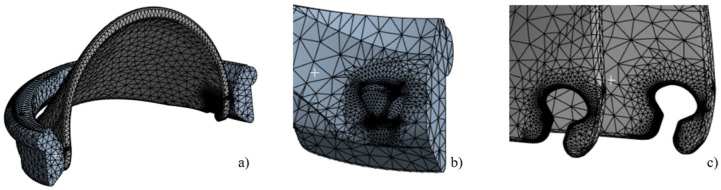
3D mesh visualization for the iValve model includes: (a) a half-model showcasing the housing and leaflet, (b) detailed hinges appendages on the housing, and (c) precise hinge sockets integrated into the leaflets for functionality demonstration.

The 3D FEA was conducted on ANSYS Static Structural. A mesh independence study was conducted using varying mesh sizes, ranging from 0.2 to 1 mm. Based on the evaluation of mesh quality, skewness, and the impact of mesh size on stress distribution and deformation, a 0.5 mm mesh size was selected for this study. To further enhance accuracy, mesh refinement was applied specifically in the contact region, as demonstrated in Figure 6, resulting in 104,138 tetrahedral elements.

**Figure 7.**
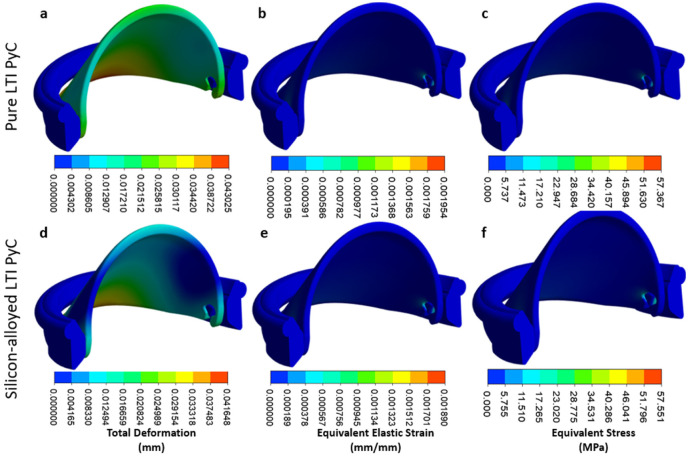
Contours are shown for total deformation, equivalent elastic strain, and equivalent stress for pure LTI PyC (a–c) and silicon-alloyed LTI PyC (d–f). These contours illustrate the material responses under applied conditions, highlighting differences in mechanical performance due to silicon alloying in LTI PyC.

The iValve housing is fixed, and the movement of the leaflet is restricted to simulate the most demanding operational conditions, ensuring the design can withstand a worst-case scenario. For Pure LTI PyC material, the analysis shows a maximum deformation for both the leaflet and housing to be 0.043 mm, an equivalent elastic strain of 0.00195 mm/mm, and a Von Mises stress of 57.37 MPa. In the case of silicon-alloyed LTI PyC, the results reveal a slightly lower maximum deformation of 0.042 mm, an equivalent elastic strain of 0.00189 mm/mm, with a Von Mises stress of 57.55 MPa. These findings demonstrate that both Pure LTI PyC and silicon-alloyed LTI PyC are mechanically robust and suitable for the proposed application, providing adequate strength and durability under stress. Figure 7 illustrates the distribution of total deformation, equivalent elastic strain, and equivalent stress for both materials, highlighting their similar performance profiles.

**Figure 8.**
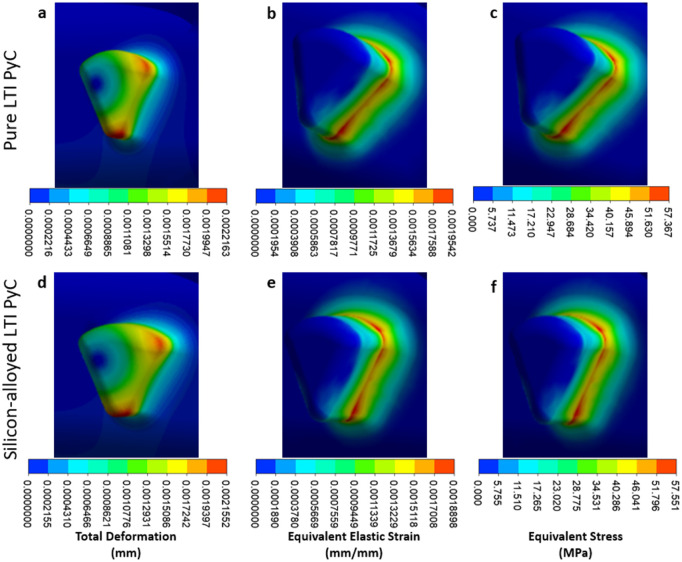
Close-up views of the housing hinge area illustrate total deformation, equivalent elastic strain, and equivalent stress for two materials: pure LTI PyC (a–c) and silicon-alloyed LTI PyC (d–f). These detailed contours provide comparative insights into the mechanical response and stress distribution of the materials under identical loading conditions.

In the housing region, the maximum deformation, equivalent elastic strain, and Von Mises stress for Pure LTI PyC are 0.0022 mm, 0.00195 mm/mm, and 57.37 MPa, respectively. For silicon-alloyed LTI PyC, these values are 0.0022 mm, 0.00189 mm/mm, and 57.55 MPa, respectively. The highest stress concentration occurs at the connection between the housing and the leaflet, indicating this is a critical area for structural integrity. Figure 8 presents the contours of total deformation, equivalent elastic strain, and equivalent stress in the housing region for both materials, providing a visual comparison of their mechanical behavior under load.

**Figure 9.**
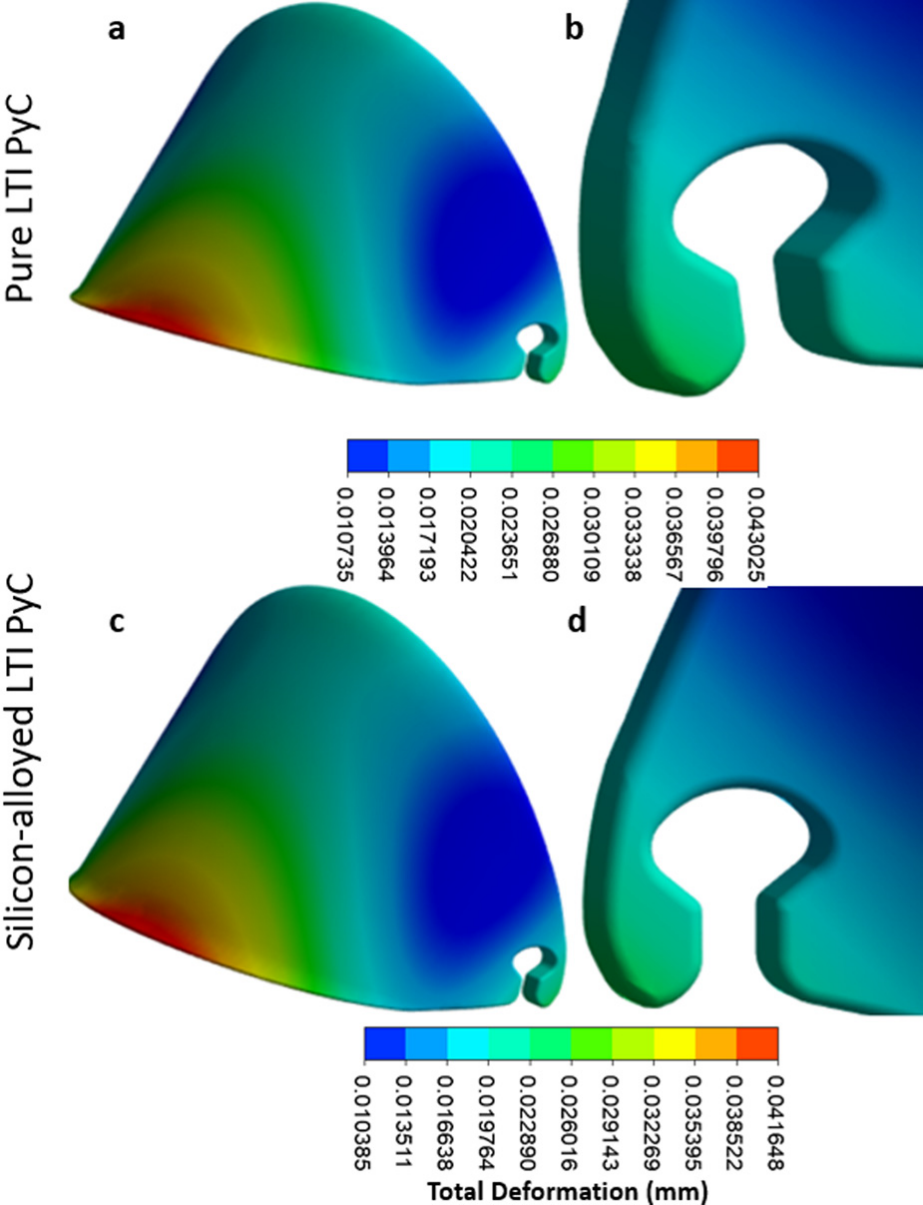
The contours for total deformation highlight the mechanical performance of two materials under identical conditions: pure LTI PyC (a and b) and silicon-alloyed LTI PyC (c and d). These visual representations provide a detailed comparison of deformation patterns, showcasing the structural response differences between the pure and silicon-alloyed variants of LTI PyC.

**Figure 10.**
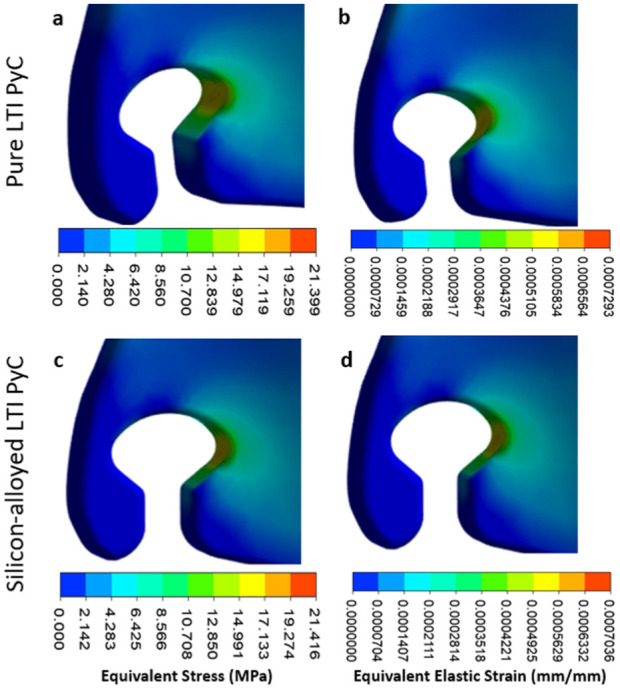
Contours for equivalent stress and elastic strain are presented for pure low-temperature isotropic (LTI) pyrolytic carbon (PyC) in panels (a and b), and for silicon-alloyed LTI PyC in panels (c and d). These visualizations illustrate the mechanical response and deformation characteristics, providing a comparative analysis of the two materials under identical loading conditions.

In the leaflet region, the maximum deformation, equivalent elastic strain, and Von Mises stress for Pure LTI PyC are 0.043 mm, 0.00073 mm/mm, and 21.40 MPa, respectively. For silicon-alloyed LTI PyC, these values are slightly lower at 0.042 mm, 0.00070 mm/mm, and 21.42 MPa, respectively. The highest stress is concentrated at the junction between the housing and the leaflet, highlighting this area as critical for mechanical performance. Figures 9 and 10 illustrate the contours of total deformation, equivalent elastic strain, and equivalent stress in the leaflet region for both materials, offering a detailed comparison of their structural behavior under stress.

A sensitivity analysis was also performed to analyze the impact of pressure change on the maximum deformation and equivalent stress. The analysis confirmed a linear relationship between the increase in applied pressure to an increase in deformation and stress. No abnormalities in stress distribution or deformation were observed with the change in pressure.

The iValve is designed to achieve effective orifice area EOA comparable to or exceeding that of the On-X valve projected at greater than or equal to 1.8 cm^2^ for a 23 mm size based on CFD simulations of steady and pulsatile flow. Unlike On X which uses a rigid pivot hinge the iValve features a low-profile recessed hinge that minimizes flow obstruction and promotes central washout enhancing EOA and reducing thrombogenic risk. Regarding valve kinematics while our current FEM study uses a quasi-static approach to evaluate peak stresses under representative loading validated against in vitro pressure data the opening and closing motions have been verified experimentally using a pulse duplicator system with high-speed imaging. These tests confirm full leaflet opening approximately 75°–80° and rapid symmetric closure within physiological timeframes less than 80 ms consistent with ISO 5840 requirements. We acknowledge that a full dynamic fluid structure interaction fluid-structure interaction (FSI) simulation would provide deeper insight into transient behavior. However, such modeling is computationally intensive and was beyond the scope of this initial durability focused study. Our combined experimental validation and quasi static FEM approach provides high confidence in functional performance. Future work will include dynamic FSI to refine leaflet dynamics.

The structural assessment of the three iValve BMHV hinge geometries, compared to two conventional BMHV hinge designs, reveals an enhanced stress distribution, leading to reduced stress concentrations in the iValve models. This optimized stress distribution is crucial, as it can significantly lower the risk of fracture and validate the structural integrity of the iValve’s hinge geometries. This preliminary validation provides confidence in the design, allowing for progression to fabrication and experimental structural testing. Additionally, it sets the stage for conducting more detailed FEA to further refine and verify the performance of the iValve.

The 3D FEA of the iValve structure offers further insights into its structural performance, with a particular emphasis on the open hinge design. As the hinge mechanism is a novel feature, it is essential to confirm its structural reliability under simulated physiological conditions. The summarized findings from this analysis, which are detailed in Table 2, underscore the importance of validating the integrity of this mechanism to ensure long-term durability.

**Table 2.** Three-dimensional finite element analysis results for iValve housing and leaflet.

Part	Material	Total deformation max. (mm)	Equivalent elastic strain max. (mm/mm)	Equivalent stress max. (MPa)	Equivalent plastic strain max. (mm/mm)
Housing	Pure LTI PyC	0.0022	0.00195	57.37	0
Housing	Si-Alloyed LTI PyC	0.0022	0.00189	57.55	0
Leaflet	Pure LTI PyC	0.043	0.00073	21.40	0
Leaflet	Si-Alloyed LTI PyC	0.042	0.00070	21.42	0

In terms of material performance, the flexural strength of pure LTI PyC is 493.7 MPa, while silicon-alloyed LTI PyC is 407.7 MPa. When comparing the maximum equivalent stresses of each component to these flexural strengths, the pure LTI PyC experiences maximum equivalent stresses for the housing and leaflet that are 11.62% and 4.33%, respectively, of the flexural strength. For the silicon-alloyed LTI PyC, the housing and leaflet see maximum equivalent stresses of 14.12% and 5.25%, respectively. These values fall well below the flexural strength limits, indicating that the components are unlikely to yield under typical working conditions.

Additionally, no plastic strain was observed in any of the components due to all simulations being consistently below yield stress, further validating the structural robustness of the iValve. Therefore, it is reasonable to conclude that the iValve, particularly its innovative hinge mechanism, is structurally sound and unlikely to fail under normal operational stresses. To advance this work, experimental fatigue testing using an accelerated wear tester is recommended, with prototypes fabricated from biocompatible materials approved for implantation. Despite the quasi-static nature of the current evaluation, which does not fully represent the complex dynamics of the cardiac environment, the findings demonstrate encouraging structural integrity of the iValve and warrant comprehensive transient dynamic and FSI studies.

## Conclusions

This study shows that the iValve’s three hinge geometries significantly improve stress distribution compared to two conventional bileaflet BMHVs, reducing stress concentrations and lowering fracture risk, validating the open-hinge concept for advancement to hydrodynamic testing. Complementary steady-state flow visualization revealed effective hinge washing during antegrade flow, with no stagnation observed, unlike in conventional BMHVs. The iValve’s design promotes continuous blood flow through the hinge socket, potentially reducing shear-induced blood damage and thrombogenicity, which may support reduced or even eliminated anticoagulation therapy. Quasi-static FEA confirmed that geometric refinements successfully mitigate stress hotspots, a key factor in long-term fatigue failure of pyrolytic carbon components. Although dynamic and high-cycle fatigue testing remain future steps, the current stress reduction provides a strong foundation for enhanced durability. The steady-state flow method enabled direct observation of intravalvular streamlines through sectioned models, offering a controlled baseline for hinge performance; future work will transition to pulsatile flow for greater physiological relevance. Based on these findings, we propose four design principles for next-generation BMHVs: Optimal Hinge Washing, ensure hinge regions are fully exposed to mainstream flow to prevent stagnation. Nodal Contact, use discrete leaflet-to-housing contact points to minimize blood cell damage and wear. Minimized Contact Forces, reduce forces during opening and closing to extend hinge life. Soft Closure Mechanism, enable gentle leaflet seating to lower impact stress, regurgitation, and backflow spikes. Fabrication in medical-grade LTI pyrolytic carbon will allow in vitro fatigue testing and eventual in vivo validation. If confirmed, the iValve’s dual advantages, superior structural durability and enhanced hemocompatibility, could pave the way for the first truly anticoagulation-free mechanical heart valve.

## References

[1] Gott VL Alejo DE Cameron DE . Mechanical heart valves: 50 years of evolution. Ann Thorac Surg 2003; 76: S2230–S2239. 14667692 10.1016/j.athoracsur.2003.09.002

[2] DeWall RA Qasim N Carr L . Evolution of mechanical heart valves. Ann Thorac Surg 2000; 69: 1612–1621. 10881865 10.1016/s0003-4975(00)01231-5

[3] U.S. National Library of Medicine. PILot Aortic Triflo Valve Study (PILATUS). https://classic.clinicaltrials.gov/ct2/show/NCT06119607 (2023, accessed 6 June 2024).

[4] Scotten LN Siegel R . Importance of shear in prosthetic valve closure dynamics. J Heart Valve Dis 2011; 20: 664–672. 22655497

[5] Yun BM Wu J Simon HA , et al. A numerical investigation of blood damage in the hinge area of aortic bileaflet mechanical heart valves during the leakage phase. Ann Biomed Eng 2012; 40: 1468–1485. 22215278 10.1007/s10439-011-0502-3

[6] Harris C Croce B Cao C . Tissue and mechanical heart valves. Ann Cardiothorac Surg 2015; 4: 399. 26309855 10.3978/j.issn.2225-319X.2015.07.01 PMC4526499

[7] Westaby S Van Nooten G Sharif H , et al. Valve replacement with the ATS open pivot bileaflet prosthesis. Eur J Cardio-Thorac Surg 1996; 10: 660–665. 10.1016/s1010-7940(96)80382-4 8875175

[8] Ihlen H Mølstad P Simonsen S , et al. Hemodynamic evaluation of the Carbomedics prosthetic heart valve in the aortic position: Comparison of noninvasive and invasive techniques. Am Heart J 1992; 123: 151–159. 1729819 10.1016/0002-8703(92)90759-o

[9] Ely J Emken M Accuntius J , et al. Pure pyrolytic carbon: preparation and properties of a new material, On-X (R) carbon for mechanical heart valve prostheses. J Heart Valve Dis 1998; 7: 626–632. 9870196

[10] Emery RW Palmquist WE Mettler E , et al. A new cardiac valve prosthesis: in vitro results. Trans Am Soc Artif Intern Organs 1978; 24: 550–556. 716055

[11] Emery RW Nicoloff DM . St. Jude Medical cardiac valve prosthesis: in vitro studies. J Thorac Cardiovasc Surg 1979; 78: 269–276. 459535

[12] Mohammadi H Fradet G . Oval housing for the St. Jude Medical bileaflet mechanical heart valve. Proc Inst Mech Eng H 2017; 231: 982–986. 28754075 10.1177/0954411917719742

[13] Goode D Dhaliwal R Schmidt J , et al. A novel approach to flow visualization through mechanical heart valves. Proc Inst Mech Eng H 2025; 239: 584–590. 40566701 10.1177/09544119251342868 PMC12209540

[14] Govindarajan V Udaykumar HS Chandran KB . Flow dynamic comparison between recessed hinge and open pivot Bi-leaflet heart valve designs. J Mech Med Biol 2009; 9: 161–176. 19865586 10.1142/S0219519409002912 PMC2768291

[15] Wurzinger LJ Opitz R Blasberg P , et al. Platelet and coagulation parameters following millisecond exposure to laminar shear stress. Thromb Haemost 1985; 54: 381–386. 2934855

[16] Mohammadi H Ahmadian MT Wan WK . Time-dependent analysis of leaflets in mechanical aortic bileaflet heart valves in closing phase using the finite strip method. Med Eng Phys 2006; 28: 122–133. 15946890 10.1016/j.medengphy.2005.03.013

[17] Mohammadi H Klassen RJ Wan WK . A finite element model on effects of impact load and cavitation on fatigue crack propagation in mechanical bileaflet aortic heart valve. Proc Inst Mech Eng H 2008; 222: 1115–1125. 19024159 10.1243/09544119JEIM432

